# Organizational Perspectives on Technical Capabilities and Barriers Related to Pediatric Data Sharing and Confidentiality

**DOI:** 10.1001/jamanetworkopen.2022.19692

**Published:** 2022-07-01

**Authors:** Michael Bedgood, Cynthia L. Kuelbs, Veena G. Jones, Natalie Pageler

**Affiliations:** 1 Department of Pediatrics, Stanford University School of Medicine, Palo Alto, California; 2 Information Services, Stanford Children’s Health, Palo Alto, California; 3 Department of Pediatrics, University of California, San Diego, La Jolla; 4 Information Management Division, Rady Children’s Hospital, San Diego, California; 5 Clinical Leadership Team, Sutter Health, Sacramento, California; 6 Palo Alto Medical Foundation Research Institute, Palo Alto, California

## Abstract

This qualitative study describes results from a semistructured interview investigating technical capabilities and barriers related to pediatric data sharing and confidentiality that was administered to health care organizations participating in the California Pediatric Informatics Collaborative.

## Introduction

Based on the Information Blocking Final Rule of the 21st Century Cures Act, the Office of the National Coordinator for Health Information Technology mandated that patients have immediate and electronic access to portions of their medical data.^1^ In pediatric care, distinct challenges to information sharing include the intertwined nature of maternal and newborn birth history, medical child abuse, differential proxy access for guardians of children, and varying state laws on adolescent consent and confidentiality rights.^2,3,4^

To address the challenges of pediatric data sharing, pediatric informaticists within the state of California created a statewide pediatric work group called the California Pediatric Informatics Collaborative. This research letter describes the formation and activities of this work group and provides results from a semistructured interview investigating final decisions of participating health care organizations, their technical capabilities, and barriers to implementation.

## Methods

This qualitative study conducted semistructured interviews via video teleconference between April 7 and September 30, 2021. The study was approved by Stanford University with a waiver of informed consent because it was considered non–human participant research. This study followed the Standards for Reporting Qualitative Research (SRQR) reporting guideline for qualitative studies.

The California Pediatric Informatics Collaborative comprises 16 organizations that provide care to children and adolescents. The unified goal of the work group is to develop strategies to share pediatric health data with patients and their proxies while appropriately protecting patient confidentiality per state minor consent laws.^5,6^ The work group met monthly using a semistructured open forum that included presentations by individual members who elaborated on innovative methods for electronic sharing of information.

One of the authors (M.B.) conducted the interviews and performed the data analysis. Questions were divided into general topics regarding portal configuration (including configurations for children aged <12 years and adolescents aged 12-17 years), ability to technically parse data elements to block access, and implementation challenges (eMethods in the Supplement).

## Results

Our study had an 81% response rate (13 of 16 organizations). Final participants included 5 stand-alone children’s hospitals, 3 large integrated delivery networks, 3 safety-net health care systems, 1 community health care system, and 1 federally qualified community health center. All 13 participants agreed about which information should be electronically released to pediatric patients younger than 12 years and their proxies in an ideal state of data sharing. The implemented portal release patterns were compared for adolescents and their proxies (Figure). Within all content (including sensitive content), variance was observed in the components that organizations could technically segment, with 12 organizations (92%) creating a sensitive note type, 11 (85%) segmenting sensitive laboratory studies, and 8 (62%) withholding sensitive imaging results. Segmentation of sensitive problems (6 organizations [46%]), medications (2 organizations [15%]), and immunizations (1 organization [8%]) was lower. All institutions recognized technical infeasibility as a barrier and were only able to electronically release a partial list of sensitive and nonsensitive content from each category. We identified 6 crosscutting themes and challenges to implementation (Table).

**Figure.**
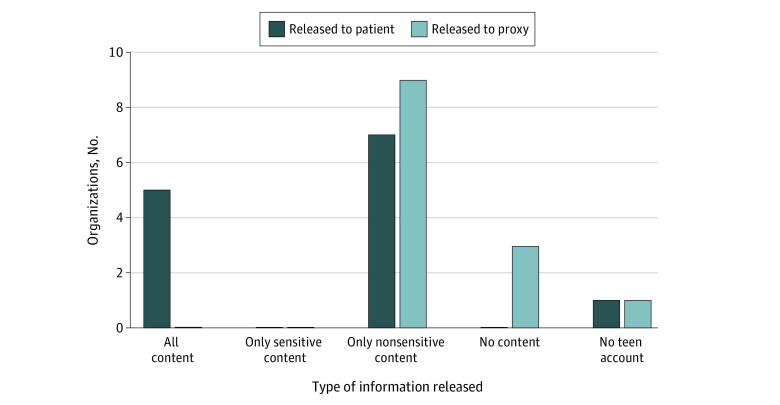
Pattern of Implemented State Release to Portal Accounts of Adolescents and Their Proxies

**Table.** Recurrent Themes Identified as Barriers to Implementation and Associated Challenges

Theme	Challenges
Technical infeasibility	Inability to segment components of data, such as specific laboratory results, medications, or problems, on a granular level when they are deemed confidential. Inability to set different release patterns for the patient and the patient’s proxy. EHR vendors provide support for interpreting law but have not yet released many technical build upgrades.
Sensitive information leak	Understanding where pitfalls are in protecting sensitive information, such as smart links pulling an extensive list of medications or problems into the EHR note, which may unknowingly release confidential information. Billing information is difficult to protect for health care confidentiality. Confidential information could be incidentally shared with third party applications, then passed along to patients’ proxies. Sensitive maternal history relevant to a newborn may be accessed by the patient or a proxy.
Permissive release of information	Ability to turn on sharing of confidential information when the adolescent patient has provided informed consent to do so.
Concern for inappropriate proxy access	Concern that adolescent patients’ accounts are being accessed by parents or patients’ proxies. Workflows are needed that will allow deactivation of adolescent accounts when it appears that the proxy has control of information. Institutions must start over in enrolling adolescents and their proxies to verify account holders more accurately.
Cultural awareness and education	Continued education of health care professionals about laws surrounding confidentiality and training on workflows for documentation.
Legal complexity	Clarification of what a psychotherapy note in the EHR can and cannot include, guided by state laws. Local interpretation of the law by independent organizations varies.

Abbreviation: EHR, electronic health record.

## Discussion

The creation of the California Pediatric Informatics Collaborative promoted early collaboration among important stakeholders across the state with regard to deciphering the 21st Century Cures Act final rule in the context of California state laws on adolescent consent. Participating organizations praised the sharing of both the general approach to data sharing and specifics about the technical capabilities of the data sharing portal. Our interviews suggested that participating health care institutions in California agreed about which information should be released to patients younger than 12 years, adolescent patients, and patients’ proxies. However, given difficulties with data segmentation and technical infeasibility, no institution, regardless of organizational type, has yet to successfully implement its entire ideal state of data sharing.

Study limitations include a predominance of Epic Systems electronic health records and difficulties in ascertaining granular details of confidential data sharing. Continued collaboration is necessary to identify a unified approach to health information sharing that will empower patients while protecting adolescent confidentiality. The creation of novel technical capabilities by electronic health record vendors to allow granular segmentation of data release will be important in this process. Future research is warranted to address the technical limitations of pediatric information sharing.

## Notes

Supplement. **eMethods.** Semistructured Interview Guide

## References

[1] Tripathi M, Posnack S. A new day for interoperability—the information blocking regulations start now. Health IT Buzz, Office of the National Coordinator for Health Information Technology. April 5, 2021. Accessed April 8, 2021. http://www.healthit.gov/buzz-blog/information-blocking/a-new-day-for-interoperability-the-information-blocking-regulations-start-now

[2] Pageler NM, Webber EC, Lund DP. Implications of the 21st Century Cures Act in pediatrics. Pediatrics. 2021;147(3):e2020034199. doi:10.1542/peds.2020-034199 33293349

[3] Office for Civil Rights. Does the HIPAA privacy rule allow parents the right to see their children's medical records? US Department of Health and Human Services. December 19, 2002. Accessed October 17, 2017. https://www.hhs.gov/hipaa/for-professionals/faq/227/can-i-access-medical-record-if-i-have-power-of-attorney/index.html

[4] Carlson J, Goldstein R, Hoover K, Tyson N. NASPAG/SAHM statement: the 21st Century Cures Act and adolescent confidentiality. J Pediatr Adolesc Gynecol. 2021;34(1):3-5. doi:10.1016/j.jpag.2020.12.015 33485521

[5] Guttmacher Institute. An overview of consent to reproductive health services by young people. Guttmacher Institute; 2020. Updated May 1, 2022. Accessed September 21, 2020. https://www.guttmacher.org/state-policy/explore/overview-minors-consent-law

[6] Duplessis V, Goldstein S, Newlan S. *Understanding Confidentiality and Minor Consent in California: An Adolescent Provider Toolkit*. 2nd ed. Adolescent Health Working Group, California Adolescent Health Collaborative; 2010. Accessed September 16, 2021. http://www.publichealth.lacounty.gov/dhsp/Providers/toolkit2.pdf

