# Supplementary material for: Organizational Perspectives on Technical Capabilities and Barriers Related to Pediatric Data Sharing and Confidentiality
Source: JAMA Netw Open. 2022 Jul 1;5(7):e2219692. doi: 10.1001/jamanetworkopen.2022.19692 (PMC9250046; doi:10.1001/jamanetworkopen.2022.19692)
Supplement: Supplement. — eMethods. Semistructured Interview Guide [file jamanetwopen-e2219692-s001.pdf]

Supplementary Online Content

Bedgood M, Kuelbs CL, Jones VG, Pageler N. Organizational perspectives on technical capabilities and barriers to pediatric data sharing and confidentiality. *JAMA Netw Open*. 2022;5(7):e2219692. doi:10.1001/jamanetworkopen.2022.19692

eMethods. Semistructured Interview Guide

This supplementary material has been provided by the authors to give readers additional information about their work.

© 2022 Bedgood M et al. *JAMA Network Open*.

eMethods. Semistructured Interview Guide

Questions About Portal Configuration

1. Which EHR vendor do you use?
    a. Cerner
    b. EPIC
    c. Other
2. Are you technically able to segment out sensitive information (notes, labs, etc) from electronic release?
    a. Yes
    b. No
    c. Unsure
3. If yes, what information are you able to segment out?
    a. Sensitive Labs
    b. Sensitive Imaging
    c. Sensitive Immunizations
    d. Sensitive Medications
    e. Sensitive Notes
    f. Sensitive Problems
4. Are you technically able to create proxy access for children with diminished capacity?
    a. Yes
    b. No
    c. Unsure
5. Have you implemented access to proxies with children with diminished capacity?
    a. Yes
    b. No
    c. Unsure
6. Do you allow providers to withhold information to a patient or others?
    a. Yes
    b. No
    c. Unsure
7. What workflow have you implemented to withhold information to a patient or others?
    a. Do Not Share Button
    b. Unshared Note Types
    c. Unshared Author types
    d. Unshared Departments

© 2022 Bedgood M et al. *JAMA Network Open*.

Questions about Children < 12 years old

The following questions will reference an "ideal state' which refers to a build that you would prefer regardless of institutional compliance or technical infeasibility.

1. In an ideal state, which of the following would you release to guardians of children < 12 years old?
    a. Demographics
    b. Immunizations
    c. Allergies
    d. Labs
    e. Medications
    f. Imaging
    g. Notes
2. In your implemented state, which of the following do you release to guardians of children < 12 years old?
    a. Demographics
    b. Immunizations
    c. Allergies
    d. Labs
    e. Medications
    f. Imaging
    g. Notes

Questions about Children 12-17 years old

The following questions will reference an "ideal state' which refers to a build that you would prefer regardless of institutional compliance or technical infeasibility.

1. Are you technically able to create a teen account for sharing information electronically to the teenager?
    a. Yes
    b. No
    c. Unsure
2. Have you implemented a teen account for sharing information electronically to the teenager?
    a. Yes
    b. No
3. Are you technically able to create a teen proxy account for sharing information electronically to the proxy?
    a. Yes
    b. No
    c. Unsure
4. Have you implemented a teen proxy account for sharing information electronically to the proxy?

© 2022 Bedgood M et al. *JAMA Network Open*.

a. Yes
b. No

© 2022 Bedgood M et al. *JAMA Network Open*.

5. How do you verify teen accounts?
    a. No verification
    b. Separate email address or phone number
    c. Self-attestation
6. What is your consent process for creating a teen account?
    a. Teenager may self-assent
    b. Proxy must consent for teenager
    c. No consent
7. In an ideal state, what information would you release to the teenager?
    a. Sensitive Content
    b. Non-Sensitive Content
    c. All content
    d. No content
8. In your implemented state, what information are you sharing to the teenager?
    a. Sensitive Content
    b. Non-Sensitive Content
    c. All Content
    d. No Content
9. In an ideal state, what information would you release to the proxy/guardian of a teen?
    a. Sensitive Content
    b. Non-Sensitive Content
    c. All Content
    d. No Content
10. In your implemented state, what information are you sharing to the proxy/guardian of a teen?
    a. Sensitive Content
    b. Non-Sensitive Content
    c. All Content
    d. No Content
11. Do you have a Family Planning, Access, Care, and Treatment (FPACT) encounter for teenagers (or similar encounter)?
    a. Yes
    b. No
12. What information is released with an FPACT encounter to the teen?
    a. Sensitive Content
    b. Non-Sensitive Content
    c. All Content
    d. No Content
13. What information is released with an FPACT encounter to the proxy?
    a. Sensitive Content
    b. Non-Sensitive Content
    c. All Content
    d. No Content

© 2022 Bedgood M et al. *JAMA Network Open*.

Questions about Difficulties with Implementation

1. What barriers to implementation have you had?
    a. Technical Infeasibility
    b. Vendor Support for Build
    c. Legal Interpretation of Information Blocking
    d. Analyst Resources
2. Do you plan to change your current state to improving patient access of information and protecting privacy?
    a. Future Build Plans
    b. Hiring More Resources
    c. Waiting for Vendor
    d. Waiting for Clarification from Governing Bodies

Questions about Workgroup

1. The workgroup presented solutions that I could take back to my organization.
    a. Strongly Agree
    b. Agree
    c. Neither Agree Nor Disagree
    d. Disagree
    e. Strongly Disagree
2. The workgroup improved my understanding of the 21st Century Cures Act final rules on information blocking.
    a. Strongly Agree
    b. Agree
    c. Neither Agree Nor Disagree
    d. Disagree
    e. Strongly Disagree
3. I will continue to participate in the workgroup's future meetings.
    a. Strongly Agree
    b. Agree
    c. Neither Agree Nor Disagree
    d. Disagree
    e. Strongly Disagree

© 2022 Bedgood M et al. *JAMA Network Open*.
